# The Ecology of ‘Acroporid White Syndrome', a Coral Disease from the Southern Great Barrier Reef

**DOI:** 10.1371/journal.pone.0026829

**Published:** 2011-12-07

**Authors:** George Roff, E. Charlotte E. Kvennefors, Maoz Fine, Juan Ortiz, Joanne E. Davy, Ove Hoegh-Guldberg

**Affiliations:** 1 School of Biological Sciences, University of Queensland, St Lucia, Queensland, Australia; 2 ARC Centre of Excellence for Coral Reef Studies, The University of Queensland, St. Lucia, Queensland, Australia; 3 The Mina and Everard Goodman Faculty of Life Sciences, Bar-Ilan University, Ramat-Gan, Israel; 4 The Interuniversity Institute for Marine Science, Eilat, Israel; 5 Global Change Institute, University of Queensland, St Lucia, Queensland, Australia; Swansea University, United Kingdom

## Abstract

Outbreaks of coral disease have increased worldwide over the last few decades. Despite this, remarkably little is known about the ecology of disease in the Indo-Pacific Region. Here we report the spatiotemporal dynamics of a coral disease termed ‘Acroporid white syndrome’ observed to affect tabular corals of the genus *Acropora* on the southern Great Barrier Reef. The syndrome is characterised by rapid tissue loss initiating in the basal margins of colonies, and manifests as a distinct lesion boundary between apparently healthy tissue and exposed white skeleton. Surveys of eight sites around Heron Reef in 2004 revealed a mean prevalence of 8.1±0.9%, affecting the three common species (*Acropora cytherea*, *A. hyacinthus*, *A. clathrata*) and nine other tabular *Acropora* spp. While all sizes of colonies were affected, white syndrome disproportionately affected larger colonies of tabular Acroporids (>80 cm). The prevalence of white syndrome was strongly related to the abundance of tabular Acroporids within transects, yet the incidence of the syndrome appears unaffected by proximity to other colonies, suggesting that while white syndrome is density dependant, it does not exhibit a strongly aggregated spatial pattern consistent with previous coral disease outbreaks. Acroporid white syndrome was not transmitted by either direct contact in the field or by mucus in aquaria experiments. Monitoring of affected colonies revealed highly variable rates of tissue loss ranging from 0 to 1146 cm^−2^ week^−1^, amongst the highest documented for a coral disease. Contrary to previous links between temperature and coral disease, rates of tissue loss in affected colonies increased threefold during the winter months. Given the lack of spatial pattern and non-infectious nature of Acroporid white syndrome, further studies are needed to determine causal factors and longer-term implications of disease outbreaks on the Great Barrier Reef.

## Introduction

Outbreaks of coral diseases have dramatically increased in recent years [1], with an exponential increase in the number of diseases documented since the first report in 1965 [2]. This increase includes the appearance of new, previously uncharacterised diseases, re-emergence of more virulent forms of known diseases, and an increase in the taxonomic range of corals affected [1], [2], [3]. Although the terms ‘disease’ and ‘syndrome’ are frequently used interchangeably within the literature (e.g. [4]), recent debates have highlighted the lack of knowledge of the underlying etiology associated with emergent diseases [2], with confusion between generalised stress responses to abiotic factors and true pathogenic diseases (e.g. [5])

The ephemeral nature of coral diseases [1], broad spatial and temporal scales of disturbances in reef ecosystems [6] and lack of quantitative data on the synergistic effects of environmental and anthropogenic stressors [7] confounds the study of disease outbreaks in coral reef ecosystems. While disease has been postulated to have a significant impact on coral populations [8], resulting in significant declines in live coral cover [9], prevalence alone is a poor indicator of disease dynamics. The impact of coral disease on community structure and function is likely to vary depending on the life history processes and the level of population affected [e.g. 10]. Most ecological surveys of disease to date have been limited to correlative observations (e.g. [4], [11], [12], [13], [14], [15]) with few studies elucidating the effects on population structure [e.g. 16] spatial pattern [e.g. 17] or modes of transmission [e.g. 18].

While a few isolated reports of coral disease exist at several locations in the Indo-Pacific during the 1980's [19], [20], coral disease research throughout the region has been largely overlooked until the past decade [2]. Regional surveys of the Great Barrier Reef conducted between 1998–2003 suggests that a wide range of disease-like syndromes are a readily identifiable phenomenon, and that a wide range of scleractinian corals are affected [21]. The most striking increase in prevalence was in ‘white syndrome’, a collective term for describing disease like syndromes showing ‘white symptoms’ [21], analogous to previously documented Caribbean diseases [see 22 for review]. The dynamics of ‘white syndrome’ are currently unclear at local scales, but spatial analysis suggests that thresholds of thermal stress and coral cover are critical factors in determining the regional prevalence of the syndrome [23].

Regional patterns of white syndrome outbreaks [23], [24], [25], [26], [27], [28] highlight a significant cause for concern, yet substantial knowledge gaps exist regarding the ecological processes and interactions across spatial and temporal scales, and little is known about the spatial pattern or impact of white syndrome at local scales. Following surveys of coral communities at Heron and Wistari Reefs (Southern GBR) in the Austral summer of 2004, large numbers of colonies of tabular *Acropora* spp. exhibited macroscopic field signs of a readily identifiable and rapid pattern of tissue loss, since termed ‘Acroporid white syndrome’ [29], [30], [31]. Tabular Acroporids are a dominant growth form in upper reef slopes across Indo-Pacific reefs [32], often forming low diversity stands [33] by actively out-competing other corals for space [34]. This study was conducted with three main objectives: (1) to determine the prevalence, spatial pattern and the impact of ‘Acroporid white syndrome’ on the community structure at Heron Reef (Capricorn Bunker group, Southern GBR), (2) to characterise the field signs and transmission associated with the syndrome, (3) to quantify rates and patterns of lesion progression in affected colonies.

## Materials and Methods

### Spatial patterns of Acroporid white syndrome

**Figure 1:**
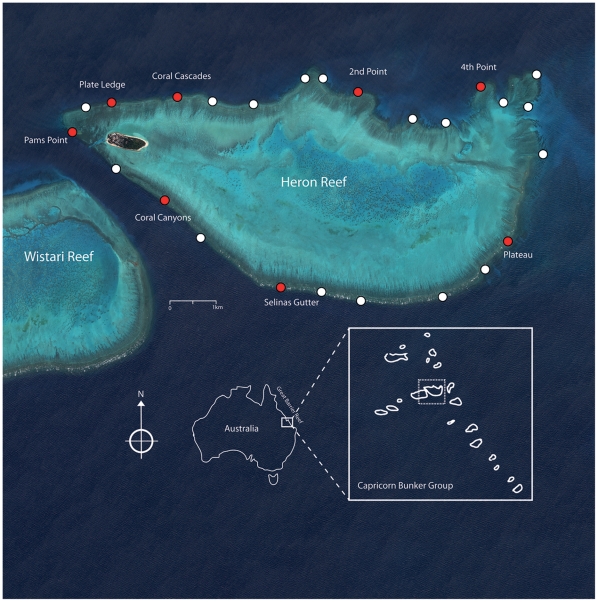
Location and regional setting of Heron and Wistari Reefs (Capricorn Bunker Group, Southern Great Barrier Reef). White circles indicate initial broad-scale sites surveyed for white syndrome presence (*n = *25), red circles indicate belt transect surveys for community structure and white syndrome prevalence (*n = *8).

To determine the broader distribution of Acroporid white syndrome at Heron Reef, 25 sites were surveyed at regular (∼1 km intervals) around the reef slope (Figure 1). Quantitative broad-scale surveys were conducted through timed swims (2 mins) between 0–5 m depth along the upper reef slope at each site, and the presence/absence of white syndrome in tabular Acroporids was recorded following the previously described macroscopic field signs [29], [30]. Following initial surveys, eight sites were selected (Figure 1). Surveys were conducted at a depth of 4–6 m on the reef slope, where the diversity and abundance of tabular Acroporids was greatest. At each site, three 40 m transects were set parallel to the reef slope, and quadrats (1 m^2^) were photographed on SCUBA using digital photography (Olympus C5060) at 1 m intervals to form 120 contiguous quadrats in total per site.

**Figure 2:**
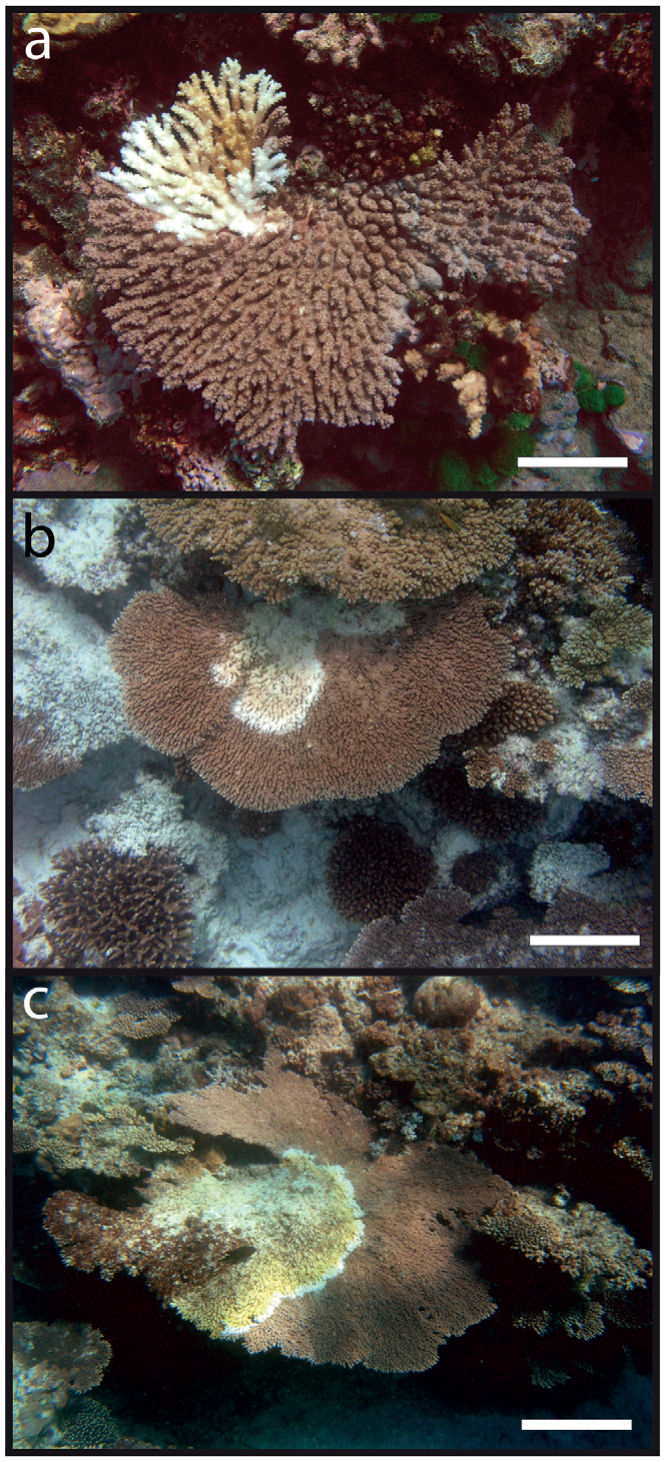
Acroporid White Syndrome in Tabular *Acropora* spp. a) size class I [0–80 cm] (Scale = 5 cm) b) class II [80–160 cm] (Scale = 25 cm), c) class III [>160 cm] (Scale = 50 cm).

The number of autonomous colonies affected by Acroporid white syndrome within each transect was recorded, and prevalence was expressed as the proportion of affected colonies within the total population of tabular Acroporids at each site. Colonies were measured from photographs across the broadest width using image analysis software (Image Tool v3.0, UTHSCA) and delineated into three categorical size classes: Class I (0–80 cm, Figure 2a), Class II (80–160 cm, Figure 2b) and Class III (>160 cm, Figure 2c) following Hughes [35]. To determine the population structure of tabular A*cropora* spp. between sites and the impact of white syndrome on size frequency distributions, the mean colony width, maximum colony size and skewness was calculated and compared across sites and between healthy and white syndrome populations using one-way Analysis of Variance (ANOVA), where assumptions of normality were tested with a Shapiro-Wilke test [36] and heteroscedasticity with Levenes test [36]. Similarity between size frequency distributions was compared using Spearmans Rank correlation co-efficient, and significant differences determined using a Mann-Whitney *U*-test [36].

To detect patterns of spatial aggregation, the number of affected colonies per transect was compared to a Poisson distribution using a G-test, with a Williams's correction factor to avoid type I error [16], [36]. The degree of dispersion of affected tabular Acroporids within transects was tested using the extra dispersion statistic [9]. Community structure and coral cover within sites was determined using the image analysis software Coral Point Count v2.6 [37]. To determine the relationship between community structure and disease prevalence within sites, coral cover was categorised according to common coral families (Acroporidae, Pocilloporidae, Faviidae, Poritidae, and ‘Other’), with Acroporidae further separated into tabular morphology [38] and other growth forms.

### Transmission and infection of Acroporid white syndrome

Infection experiments were conducted in aquaria to determine the pathogenicity of white syndrome. Fragments of *Acropora cytherea* (5 cm length, *n* = 164) were collected from nearby Wistari Reef (Figure 1), and artificial lesions created at the base of each fragment using a sterile scalpel. Explants were maintained in through-flow aquaria (80 *l*) at Heron Island Research Station. In order to investigate the infectivity of white syndrome at both ambient seawater temperature and under thermal stress, two separate treatments were conducted: control explants were maintained at ambient temperature (25±1 °C, *n = *100), and thermally stressed explants were subjected to elevated seawater temperature (30±1 °C, *n = *64). The seawater temperature was initially increased by 1°C per day for four days and the coral explants were then acclimated for three days at 30±1 °C prior to initiation of the experiment. Inoculates were created by using a waterpik to remove surface mucus and tissue from healthy and diseased fragments of *A.cytherea* (∼10 cm) in 20 ml of 0.2 µm filtered seawater. In each treatment corals were either inoculated with white syndrome inoculum (ambient *n = *50, heated *n = *32) or healthy inoculum (ambient *n = *50, heated *n = *32) using a syringe. Following inoculation, water flow was inhibited for 48 hrs to reduce potential dilution of inocula. Explants were monitored for 21 days for signs of tissue loss associated with the syndrome.

In-situ transmission experiments were conducted by grafting healthy fragments of *A.cytherea* onto existing colonies affected by white syndrome. Parent colonies of asymptomatic tabular *Acropora* (*n = *6) and colonies with active lesions (*n = *6) were identified and tagged at 5–8 m depth at Wistari Reef. Fragments (approximately 80 cm^2^, *n* = 24) were collected from healthy colonies of *A.cytherea*, and two fragments were grafted to the outer edge of each host colony using plastic cable ties. In white syndrome affected colonies, fragments were grafted to intact tissue immediately preceding the active lesion margin. Fragments were monitored over a period of 28 days for progression and transmission of white syndrome lesions, and to ensure continued contact between host and graft. Qualitative mortality of grafts between control and affected colonies was recorded after 28 days and a Chi-Squared analysis with Yates's continuity correction [36] was performed to identify differences in mortality between treatments.

### Temporal Dynamics of Acroporid white syndrome

To investigate the dynamics of tabular *Acropora* affected by white syndrome, a monitoring program was established at Coral Canyons on the southern edge of Heron Reef (Figure 1). This site was chosen due to an abundance of large (>2 m) and smaller colonies of tabular *Acropora* spp., and its relative proximity to Heron Island Research Station. Seawater temperatures (°C) were recorded at 30-minute intervals at 8 m depth throughout the monitoring period using *in-situ* temperature loggers (Optic Stowaway^tm^, Onset Corporation). A defined area of reef slope (80×20 m, 6–8 m depth) was surveyed on SCUBA, and colonies of tabular *Acropora* (*n = *14) with signs of active lesions were tagged using cattle tags. Additionally, colonies of healthy (asymptomatic) tabular *Acropora* within the study site were tagged to determine the incidence of novel lesions of white syndrome throughout the monitoring period.

All colonies were monitored weekly for signs of lesion progression following the Austral summer (Feb–Apr) and winter months (Jul–Sep) of 2004 by digital photography (Olympus C5060) using a series of permanent markers attached to the coral skeleton. Images were imported into Illustrator CS (Adobe Systems) and consecutively layered to form a time series, and the colony outlines and lesions were traced. The resulting images were analysed using image analysis software Matrox Inspector (v2.1, Matrox Imaging, Canada) and linear progression of lesions (cm^−1^ week^−1^), tissue loss resulting from lesion progression (cm^−2^ week^−1^) and changes in colony area were measured. The relationship between colony size, tissue area, lesion perimeter and rates of progression was examined using linear regression [36]. A t-test was used to identify differences progression rates between summer and winter [36].

## Results

### Spatial patterns of Acroporid white syndrome

**Figure 3:**
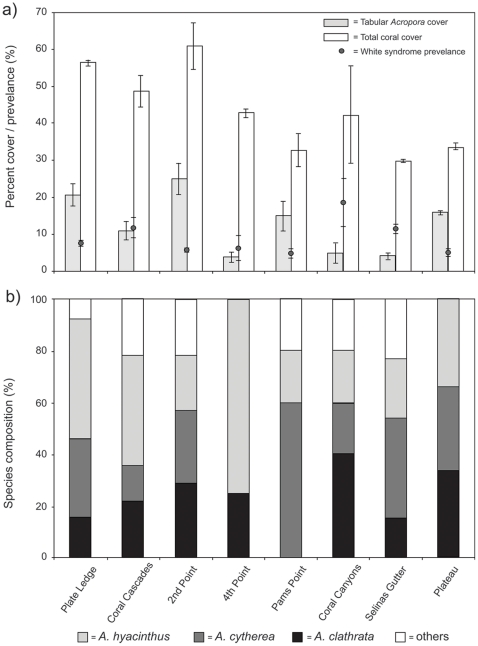
Coral community structure and Acroporid white syndrome prevalence at sites around Heron Reef: a) Coral cover and prevelance (± standard error), b) Species composition of tabular *Acropora* spp. affected by white syndrome.

White syndrome was broadly distributed across Heron Reef, and was observed to affect tabular *Acropora* spp. at all 25 sites surveyed. Although depth was not an implicit factor in these surveys, white syndrome was observed to affect tabular *Acropora* in the upper reef crest (<2 m) and solitary colonies on the lower reef slopes up to 18 m in depth. We recorded a total of 1220 colonies of tabular *Acropora* at eight sites at Heron Reef, with a mean prevalence of 8.1±0.9%. Significant differences in prevalence were observed between sites (ANOVA, *F = *3.50, p<0.05, Table 1), ranging from 4.9±1.2% (Pam's Point) to 14.3±2.5% (Coral Canyons). The structure and composition of coral communities was highly variable between sites, with total coral cover varying between 29.9–60.8% (Figure 3a). Acroporidae was observed to be the dominant family of coral, accounting for 84.2±2.9% of all coral cover. Tabular morphology was the dominant growth form of *Acropora* observed during surveys (averaging 66.3±6.6% of all *Acropora* corals), varying between 3.7% (4^th^ Point) to 25.1% coverage (2^nd^ Point). A weak but significant relationship was observed in that white syndrome was more common in sites with a higher cover of tabular *Acropora* (β =  −0.44, r^2^  = 0.2, p<0.05). This relationship was not observed for total *Acropora* cover (β =  –0.21, r^2^  = 0.04, p>0.05) or total coral cover (β =  −0.18, r^2^  = 0.03, p>0.05).

White syndrome was observed to affect the three main species of tabular *Acropora* found at the upper reef slope at Heron Reef (*A. hyacinthus*, *A. cytherea*, and *A. clathrata*, Figure 3b), as well as other less common species (*A. glauca*, *A. microclados*, *A. subulata*, *A. anthoecercis*, *A. solitaryensis*, *A. granulosa*, *A. loripes*, *A. caroliniana*, *A. selago*) that were grouped due to their relatively low abundance. The abundance of tabular colonies affected by white syndrome was strongly correlated to the abundance of tabular *Acropora* within transects (β = 0.66, r^2^  = 0.44, p<0.001), yet this relationship was strongly dependant upon mean colony size. While no significant relationship was observed between the abundance of healthy and affected colonies in smaller colonies (Class I, β = 0.06, r^2^  = 0.01, p>0.5), a strong relationship was observed in medium (Class II, β = 0.62, r^2^  = 0.39, p<0.01) and large (Class III, β = 0.62, r^2^  = 0.38, p<0.01) colonies.

**Figure 4:**
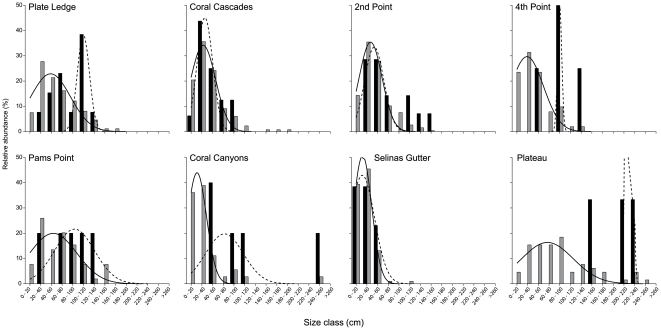
Size frequency distributions of healthy and white syndrome affected tabular *Acropora* spp. Grey columns = healthy colonies, black columns = white syndrome affected colonies. Histograms fitted with normal distributions (solid line = healthy colonies, dashed line = white syndrome affected colonies).

The population structure of tabular *Acropora* spp. varied significantly between sites (Table 1) in terms of mean colony size (50.05±4.59 cm^−1^, ANOVA *F* = 29.78, p<0.001) and maximum colony size (108.36±10.04 cm^−1^, ANOVA *F* = 7.29, p<0.001). A positive skew in colony size was observed in all sites, suggesting a greater abundance of smaller colonies of tabular *Acropora* spp. within the population (1.42±0.23, ANOVA *F* = 0.74, p>0.5). The size frequency distributions of healthy colonies were relatively homogenous (mean correlation coefficient = 0.83±0.02 SE, p<0.05) indicating a high degree of similarity in populations between sites (Figure 4), with the exception of Selina's Gutter and Plateau (correlation coefficient = 0.47, p>0.05).

The mean size of tabular *Acropora* spp. affected by white syndrome (86.8±11.89 cm) was significantly higher than that of the healthy population (ANOVA *F* = 20.73, p<0.001), although this varied between sites (ANOVA, *F* = 10.80, p<0.001, Figure 4). Although the species distribution of tabular *Acropora* spp. between sites was variable (Figure 3b), no significant difference was observed between the mean size of the main host species (*A.hyacinthus*, *A.cytherea*, *A.clathrata*, ANOVA, *F* = 1.87, p>0.1). The mean skewness of white syndrome colonies was not significantly different between sites (0.10±0.37, ANOVA, *F* = 0.27, p>0.5), although three of the sites were negatively skewed (Plate Ledge, Pam's Point and Plateau, Figure 4). Our results indicate a trend towards a negative skewness of white syndrome colonies compared to that of healthy colonies within sites (ANOVA, *F* = 9.55, p<0.01), suggesting that white syndrome disproportionately affects larger colonies of tabular *Acropora* spp. within the population (Table 1). Further, although large colonies of tabular *Acropora* spp. (Class III) were rare across all sites (0.3% of total counts), >50% were affected by white syndrome.

The frequency distribution of white syndrome colonies within each transect (40 m^2^) did not differ significantly from a Poisson distribution (G-test, Gadj = 7.77, d.f. = 7, p>0.1), suggesting that the occurrence of a colony affected by white syndrome within a transect did not increase the probability of finding a second affected colony. Further, white syndrome appears to be over dispersed in distribution (extra dispersion value λ = 0.748, p<0.01).

### Characterisation and transmission of Acroporid white syndrome

Acroporid white syndrome is characterised by a distinct line between apparently healthy tissue and exposed white skeleton in tabular *Acropora* spp., resulting from the progressive degeneration of the coenosarc and polyp bodies at the lesion boundary. No signs of tissue bleaching (paling associated with the loss of zooxanthellae) were observed in tissue preceding the lesion margin. As lesions progressed, a green band was observed in the recently denuded skeleton as a result of photo-acclimation of endolithic algae in response to the loss of shading tissue layers [31]. *In-situ* inspection and analysis of biopsy samples by light microscopy revealed an absence of apparent microbial populations or eukaryotic organisms, although several protists were found to be associated with white syndrome lesions, including a holotrich ciliate (possible *Paramecium* sp.) thought to be related with “brown band syndrome” [4], [39] and the heterotrich ciliate *Halofolliculina corallasia*, previously identified as the causative agent of “skeletal eroding band” [40].

**Figure 5:**
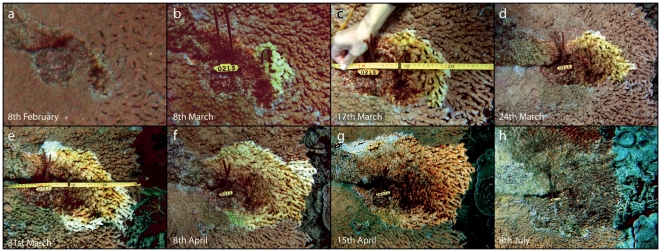
Initiation, formation and progression of white syndrome lesion in a colony of *Acropora cytherea*. Photographs taken during weekly monitoring in the summer months of 2004 (a–g) and winter (h).

**Figure 6:**
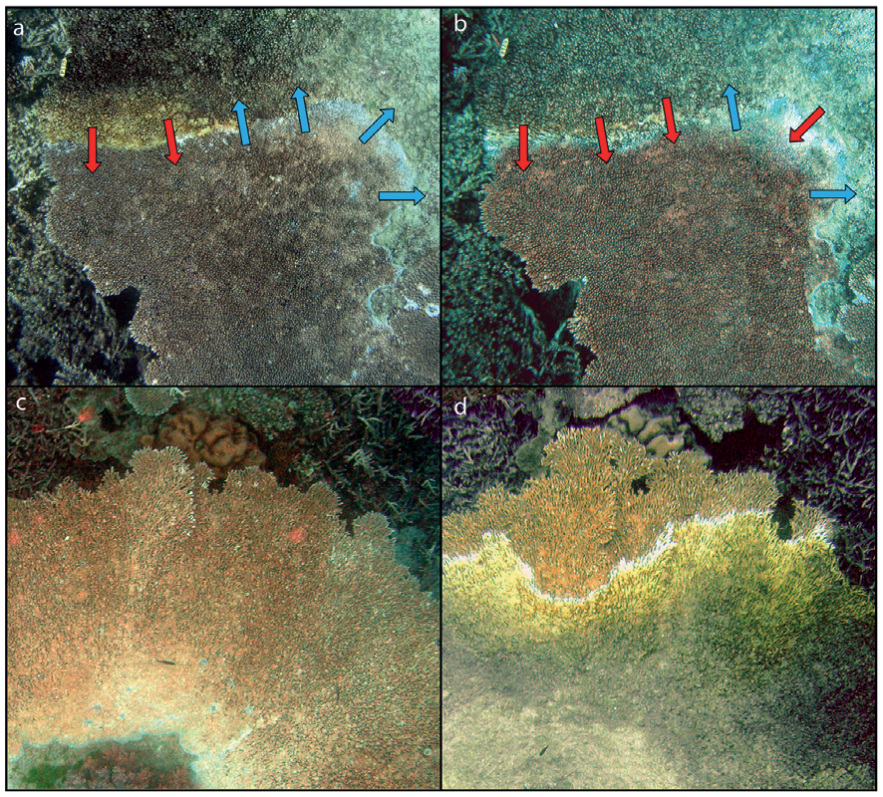
Temporal patterns of white syndrome lesions. Tissue degeneration (indicated by red arrows) and regeneration (indicated by blue arrows) in lesions from a) 6^th^ April 2004, b) 14^th^ April 2004, and evidence of axial growth of *Acropora cytherea* affected by Acroporid white syndrome in c) 6^th^ April 2004, d) 20^th^ February 2006.

In almost all (>95%) observations, white syndrome was observed to develop in a characteristic and repeatable pattern initiating from the basal margins of branches at the centre of affected colonies. Lesion formation occurs at a focal point (locus) at the proximal interface between regenerating ‘healthy’ tissue and dead skeleton (Figure 5 a), and spreads rapidly along this margin (Figure 5 b,c). Following the emergence of the initial lesion, progression occurs in a radiating pattern from the proximal region of the colony towards the outer distal regions (Figure 5 d–g). Lesions diffuse and spread outwards, forming a radial pattern of lesion progression in which tissue loss in the distal portions of the colony is several orders of magnitude greater than in the centre of colonies. Colonies affected by white syndrome lesions showed intermittent signs of regeneration, including coenosarc regrowth and active skeletal re-deposition along lesion borders (Figure 6 a,b). Multiple lesions were frequently observed to develop simultaneously and independent of another in larger colonies, often converging to form individual lesions in the latter stages of colony mortality. We observed active growth in the axial branches of colonies affected by Acroporid white syndrome throughout the monitoring period (Figure 6 c,d).

Pathogenicity experiments suggest that white syndrome is not readily transmitted in aquaria or in the reef environment. Inoculation of fragments at ambient temperatures (25±1°C) and elevated temperatures (30±1°C) resulted in no development of lesion formation or signs of tissue degeneration after 14 days post-inoculation. *In-situ* field experiments of white syndrome transmission at ambient temperatures produced similar results, with no significant difference between the levels of mortality between healthy fragments grafted to colonies affected by white syndrome or healthy colonies (Chi-Squared, χ*^2 = ^*2.71, p>0.5).

### Temporal Dynamics of Acroporid white syndrome

**Figure 7:**
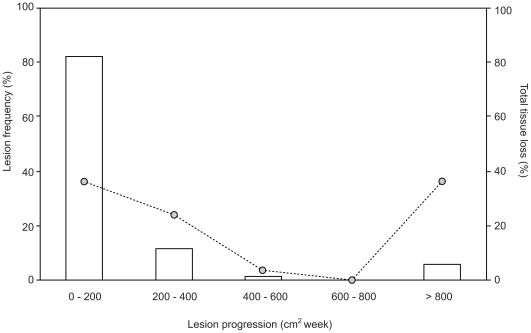
Size frequency distribution of lesion progression and tissue loss from Acroporid white syndrome colonies. Colonies monitored between March–August 2004.

Monitoring of colonies affected by white syndrome revealed high variability in rates of lesion progression, with a complex interplay between tissue degeneration and intermittent regeneration along lesion margins. White syndrome appears to be chronic within individual colonies, with active lesions observed within individual colonies over the eight months of monitoring. Although lesions were observed along the entire radius of colonies (>3 m in width), active progression was frequently inconsistent across the length of the lesion perimeter. Linear progression of white syndrome lesions was highly variable across colonies (0–13.47 cm^−1^ week^−1^) throughout the monitoring period, resulting in similarly variable high rates of tissue loss (0–1145.66 cm^−2^ week^−1^). Smaller tissue lesions (0–400 cm^−2^) where more frequently observed (93.5% observations) within affected colonies, and accounted for over half (60.3%) of total tissue mortality throughout the monitoring period. Intermediate sized lesions (400–800 cm^−2^) were less frequently recorded (1.1%) and resulted in relatively low rates of tissue mortality (3.5%). Larger lesions (>800 cm^−2^) were significantly less frequent (5.4%) yet contributed to a high proportion (36.2%) of total tissue mortality, implying that while the continuous loss of tissue resulting from such smaller lesions leads to longer term chronic effects in larger colonies (>100,000 cm^−2^), infrequent tissue loss resulting from intermediate and larger lesions has significant effects on tissue mortality at a colony level (Figure. 7).

**Figure 8:**
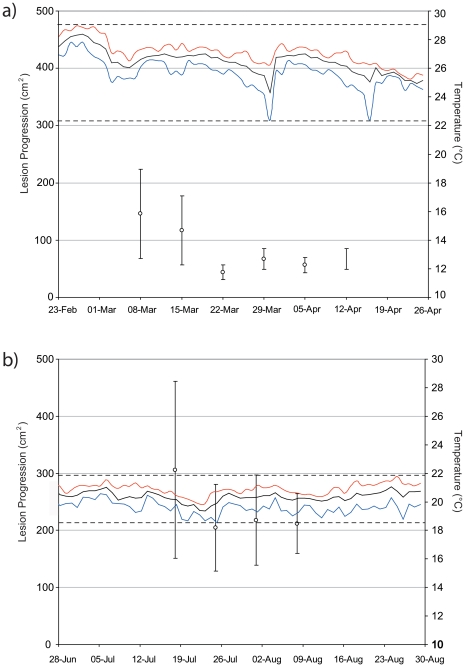
Temporal patterns of tissue loss in Acroporid white syndrome. Lesion progression (cm^−2^ ± SE) and average temperature (°C ± min/max) recorded during summer months (March–April) and winter months (July–August).

Rates of lesion spread were non-uniform and highly variable. During the summer months of 2004, sweater temperatures on Heron Reef were >27°C for several weeks (Figure 8), reaching a maximum temperature of 28.4°C. Throughout this period, rates of tissue loss from lesions averaged 90.0±17.8 cm^−2^ week^−1^ (0–917.8 cm^2^). In winter months, water temperatures were considerably cooler, reaching a minimum of 18.6°C. During this period, rates of tissue loss were threefold those recorded earlier in the year (t-test, *t* =  −3.77 p<0.001), averaging 263.2±58.6 cm^−2^ week^−1^ (10.14–1145.66 cm^2^) across colonies. Tissue loss from white syndrome does not appear to be related to intact colony area (β = 0.24, r^2^ = 0.06, p>0.1) or lesion perimeter (β = 0.21, r^2^ = 0.02, p>0.1) suggesting that rates of lesion progression within colonies is not dependant on the remaining area of intact live tissue. A small but significant relationship was observed with colony size (Linear regression, β = 0.56, r^2^ = 0.23, p<0.05) in that rates of lesion progression were greater in larger colonies.

**Figure 9:**
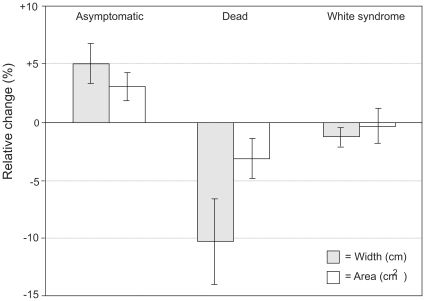
Relative change in growth in tabular *Acropora* spp. monitored during 2004.

Monitoring of healthy colonies within the site revealed an increase in the incidence of white syndrome during the summer and winter months (Figure 8), with novel lesions (*n* = 5) initiating and spreading in previously asymptomatic colonies. Throughout the monitoring period a positive linear relationship was observed in surface area (%) and linear growth (cm^−1^) in asymptomatic tabular *Acropora* (β = 0.66, r^2^ = 0.85, p<0.001), while dead colonies decreased in both width and area due to increased bioerosion (Figure 9). Colonies affected by white syndrome showed an average decrease in width and surface area (Figure 9), yet high variability was observed in linear extension rates in colonies affected by white syndrome (range =  −3.46–7.12 cm), indicating active linear growth.

## Discussion

### Spatial patterns of Acroporid white syndrome

Our results indicate that Acroporid white syndrome was wide spread across Heron Reef in 2004, affecting tabular *Acropora* spp. at all 25 sites surveyed (Figure 1). The macroscopic field signs of Acroporid white syndrome are consistent with those seen elsewhere in the Indo-Pacific region since this study was conducted [24], [26], [28], [41], suggesting that the syndrome is regional in extent. The mean prevalence observed in the present study (8.05±0.89%) is considerably higher than reported in other locations, and greater than that recorded in the year preceding our study (2003, [4]) and an order of magnitude higher than recorded in following years (<0.5%, 2007–2009 [42]). Although global comparisons of disease prevalence are inherently problematic, the highest prevalence of Acroporid white syndrome recorded in the present study (14.3±2.5%) is comparable to outbreaks of disease observed in Caribbean white syndromes [9], [43]. Our results appear to counter previous suggestions that high coral diversity in the Indo-pacific region may potentially lower the spread of disease [12].

The prevalence of Acroporid white syndrome appears relatively homogenous between sites on Heron Reef, despite widespread differences in community structure. Consistent with previous studies [24], there were no significant differences in prevalence at exposed sites (e.g. Selina's Gutter, characterised by high scleractinian diversity with low tabular *Acropora* and total coral coverage) and sheltered sites (e.g. Coral Cascades, characterised by relatively low scleractinian diversity with a high tabular *Acropora* and total coral cover). Although we recorded differences in the species composition of white syndrome colonies between sites, comparisons of species specific-prevalence were problematic, as our surveys did not record species-level identification of asymptomatic tabular *Acropora* due to the sheer abundance of colonies, and inherent difficulties of *in-situ* species identification of Acroporids [e.g. 38]. Given the potential for species-specific host resistance and the high diversity of corals of the genus *Acropora*, future studies of disease prevalence throughout the Indo-Pacific region should target the incidence of disease at both genus and species level.

Our results indicate that the abundance of tabular colonies affected by white syndrome is related to the abundance of tabular *Acropora* within transects. These results are consistent with host-density relationships in Indo-Pacific white syndrome [24], [44], [45]. Previous regional surveys of the outer reefs of the GBR have shown that coral cover is a key factor in white syndrome, where outbreaks occur following thermal stress on high coral cover reefs (>50%) such as the Capricorn Bunker groups [23]. Despite a strong link between the abundance of tabular Acroporids and white syndrome in the present study, at a local scale our results show no relationship between Acroporid white syndrome prevalence and either coral cover or total *Acropora* cover. This finding is consistent with another study of white syndrome in tabular *Acropora* spp. [24], and suggests that host-density dependence is decoupled from simple metrics of coral cover. It seems plausible that the prevalence of white syndrome in high coral cover reefs on the GBR [23] may be driven by a high abundance of tabular Acroporids rather than total coral cover.

Acroporid white syndrome exhibited a non-aggregated spatial pattern, in that the incidence of disease is unaffected by proximity to other colonies [16], [46], [47]. This finding is entirely consistent with field observations of neighbouring tabular *Acropora* colonies growing in direct proximity, where white syndrome results in entire colony mortality of one colony, yet does not transmit to adjacent (and otherwise healthy) colonies (Roff, pers.obs.). The over-dispersed distribution nature of Acroporid white syndrome also suggests that the higher rates of white syndrome in the outer shelf reefs of the Capricorn Bunker Group are not primarily due to increased ‘pathogen transmission’ in regions of higher coral cover, as previously suggested [4]. In contrast, coral diseases caused by known bacterial pathogens (such as ‘white pox’ disease, [9]) exhibit a strongly aggregated spatial pattern consistent with epidemiological models of nearest-neighbour contagion. While the role of vectors in white syndrome transmission has yet to be explored, white plague in the Caribbean exhibits a similar non-aggregated spatial pattern [16], and is at least partially dependant on algal contact to induce lesions [18].

Mortality rates are often relatively high within populations of tabular *Acropora* spp. [e.g. 48]. While previous studies have suggested that larger colonies are less susceptible to disturbance events than smaller colonies i.e. a Type III survivorship curve [49], [50], coral communities exhibit differential responses to biotic and abiotic stressors. Mass bleaching of coral populations following periods of thermal stress results in a higher survivorship of smaller colonies [51], [52], and larger colonies are more likely to be affected by epizootics of white plague disease in the Caribbean [16]. Our results indicate that although rare, larger colonies of tabular *Acropora* (>160 cm, Class III) are disproportionately affected by white syndrome. While it is tempting to implicate senescence [e.g. 53] as a key factor in the disproportionate incidence of white syndrome in larger colonies, growth of a colony as a whole is potentially indeterminate [54] as the relationship between size and age is decoupled by differential growth rates, partial mortality, competition and abiotic factors [55], [56], [57]. Alternatively, our results may merely reflect the ephemeral nature of disease in that acute rates of tissue loss can result in rapid mortality of a smaller colony, while chronic lesions may persist in larger colonies.

In terms of population dynamics, fecundity in corals is strongly size dependant, with larger colonies exhibiting disproportionately higher rates of fecundity, egg size and gonad ratio than smaller colonies [50], [58], [59]. Colonies of smaller tabular *Acropora* (Class I) are likely to be below the minimum reproductive size [50], as energy is directed towards initial colony growth [35]. The overall positive skew in the healthy population observed in this study is constant with previous population modelling of tabular *Acropora* in which recruitment rates are high [33]. With a disproportionate reduction in the number of larger colonies of tabular *Acropora* as a result of white syndrome, the fecundity of the population is dependant on continual growth (and subsequent reproductive capability) of smaller colonies and may potentially disrupt key demographic processes [55]. Increased mortality following disturbance opens up substratum for recruitment of juvenile corals by removing the shading effect of tabular *Acropora* [34], [48], potentially increasing genetic diversity [46], limited recruitment of corals following disease epizootics has been observed [46], [60]. Initial surveys of recruitment on tabular *Acropora* spp. that died during the 2003–2004 outbreak of Acroporid white syndrome at Coral Canyons indicates that recruitment rates of *Acropora* spp., are considerably higher than surrounding available substrate (Roff, unpublished data), suggesting that recovery of coral communities at these sites may be more rapid than previously assumed.

### What is Acroporid white syndrome?

The macroscopic field signs of white syndrome in tabular *Acropora* are similar to those previously reported from the Caribbean region [22], with the exception of the zone of bleached tissue preceding the lesion margin associated with white band type II [61] and white plague type II [47]. Histological and microbiological samples of tabular *Acropora* affected by white syndrome revealed no evidence of microbial communities associated with lesions, and no evidence of tissue necrosis associated with bacterial diseases of corals [62]. Failure in this study to identify white syndrome as contagious through either aquaria inoculation experiments or *in-situ* transmission experiments is consistent with the spatial patterns observed in the field, and further questions the pathogenic nature of white syndrome and the involvement of bacterial pathogens [63]. In contrast to our study, a highly contagious bacterial coral disease from the Caribbean termed white plague type II caused by *Aurantimonas coralicida* [64] is readily transmissible to healthy colonies in aquaria through indirect contact with diseased corals [43]. Although the involvement of a pathogen in the initial stages of lesions formation cannot be excluded, in established lesions pathogens appear to be largely absent from white syndrome lesions [62], and the syndrome does not appear to be infectious between hosts. Culture independent analysis of bacterial communities associated with Acroporid white syndrome at Heron Reef [63] revealed that *Vibrio* pathogens previously implicated with white syndrome in the Indo-Pacific [27] were largely absent from affected colonies, further questioning the role of bacterial pathogens as the causative agent of Acroporid white syndrome.

While the association between protists and disease lesions are well documented within literature [19], [39], [40], [65], [66], [67], the interactions between white syndrome lesions and protists is currently unclear. The infrequent incidence and absence of tissue lysis associated with the loricae of *Halofolliculina corallasia* penetrating coral tissue ahead of the lesion boundary [68] observed in the present study suggests an opportunistic association as harmless epizoism [65] rather than a synergistic pathogen of white syndrome. The causative agent of the coral disease BrB remains unknown [69], and the delineation between ‘white syndrome’ and ‘brown band’ [4] is unclear. Analysis of corals affected by the syndrome suggests that the ciliates are secondary rather than a primary cause of disease lesions [69]. Brown band ciliates have also been observed on lesions created by Crown of Thorns starfish [70], further suggesting that ‘brown band’ may be a sign of opportunistic infections of ciliates on decaying tissue rather than being implicated as a causal factor. Considering that brown band ciliates were infrequently observed in low densities on Acroporid white syndrome lesions (and were frequently absent from colonies), the presence of the ciliates on lesions may represent an opportunistic association rather than a macroscopic field sign of the syndrome.

Initiation and progression of white syndrome lesions appears to be similar to that of other band diseases [71] in that in almost all observed cases, lesions were observed to initiate from basal regions the colony. Considering such distinct patterns of lesion formation within colonies affected by white syndrome, it seems unlikely that generalised stress leading to the disruption of the holobiont [72] and subsequent shifts in microbial communities across entire colonies [73] would result in such specific patterns of lesion formation. Future research should take into consideration interactions at the base of colonies [e.g. 18,53] as a mediating factor in the formation of white syndrome. Initial observations of Antonius [71] suggest that initiation of lesions of white band disease occurred at the interface between live tissue and algal overgrowth, and further speculated that the disease may be associated with a trauma or a biochemical response from epibenthic algae rather than a bacterial pathogen [74], which is consistent with problems identifying the etiology of this disease [22], [75], [76].

### Temporal dynamics of Acroporid white syndrome

Previous monitoring of individual colonies affected by disease have either involved intensive sampling over short time scales [e.g. 9] or observations based over broader time scales with little temporal replication [e.g. 77]. The rates of lesion progression observed in this study are orders of magnitude higher than previously observed in Caribbean white syndromes [see 22 for review]. Given that corals were monitored on a weekly basis, the variable width of the white band of exposed skeleton (mm^−1^ to cm^−1^ week) suggests that a higher frequency of monitoring (e.g. daily) would likely reveal significantly greater rates of tissue loss associated with the syndrome. Longer term monitoring of colonies showed that although acute rates of tissue loss in smaller colonies resulted in entire colony mortality within several months, the syndrome was chronic in larger corals (>2 m) throughout the monitoring period, resulting in sustained levels of partial mortality. Although partial mortality do not impact upon population size as such, a reduction in the area of living tissue is likely to reduce the reproductive output of individual colonies [50]. The ability of corals to regenerate lesions is strongly dependant on both intact tissue surface area [78] and perimeter to surface area ratio of lesions [79]. In contrast, our results suggest this relationship is decoupled in that white syndrome lesions were often as wide as the colony radius (>200 cm) and lesion progression within colonies did not increase through time, even though intact colony area decreased significantly through tissue loss.

Considering broad-scale patterns of increases in white syndrome frequency following periods of thermal stress [23], the lack of strong dependency of thermal stress in lesion progression observed in the present study is intriguing. Given the significant variation in lesion formation and progression within affected colonies observed throughout the monitoring period, it is difficult to implicate temperature as direct forcing factor in the progression of white syndrome lesions. The continuous progression of lesions throughout summer and winter months is in stark contrast to ephemeral epizootics such as ‘atramentous necrosis’ from the northern GBR [80], where lesions were only visible for 3–4 weeks following the onset of thermal stress and where conspicuously absent during the winter months (although see [81]). Following the monitoring period in 2004, monitored colonies were intermittently surveyed for lesion progression throughout 2005–2008. Despite evidence of intermittent regeneration of lesion margins during the monitoring period (Figure 6), all colonies suffered complete mortality following initiation of white syndrome lesions.

### Summary and conclusions

We identify a form of coral disease from the southern Great Barrier Reef, termed Acroporid white syndrome. Based upon macroscopic field signs, the syndrome appears similar to several other cases reported around the Indo-Pacific region [24], [26]. Surveys indicate that white syndrome is prevalent across all sites surveyed at Heron Reef, and spatial patterns indicate a role of host density on the prevalence of white syndrome. Our results indicate that Acroporid white syndrome does not readily transmit between colonies in the field, and initiation and progression of lesions occurs in both summer and winter temperatures.

The temporal dynamics of white syndrome in the southern GBR between 2000 and 2010 are intriguing. The initial surveys of coral disease in the GBR indicate a peak of outbreak prevelance in 2003 [4] following a coral bleaching event in the preceding year [23]. Subsequent surveys indicate that although prevalence declined to intermediate levels between 2004–2009, the current levels remain considerably higher than when monitoring began in 1999 [42], [82]. Monitoring at the same locations as the present study (Coral Cascades, 2^nd^ Point, 4^th^ Point) show a decline in tabular *Acropora* spp between 2007–2009, resulting in a shift in community structure [42]. In 2004 we recorded high levels of coral cover at these sites (e.g. up to 25% tabular *Acropora* at Second Point), yet analysis of community structure at these sites four years following our study [42] indicates that these sites are now dominated by other growth forms of *Acropora* (2^nd^ Point) and *Faviites* (Cascades). While this may reflect slight differences in site location and spatial heterogeneity in coral community structure, whole-colony mortality following the peak of the outbreak in 2003–2004 may have resulted in (unfortunately undocumented) high rates of mortality and loss of tabular *Acropora* across Heron Reef.

The low prevalence of white syndrome in subsequent years reported by Haapkyla et al [42] is consistent with density dependence reported in the present study, in that high mortality of larger colonies following the initial outbreak in 2003–2004 resulted in a reduction in tabular Acroporid density across all sites, and a lower overall prevalence of white syndrome in tabular *Acropora* spp.. Subsequent observations across all sites between 2005–2007 indicate high densities of in-situ and eroding dead skeletons of tabular Acroporids, and all colonies monitored during 2004 at Coral Canyons were dead. Given the lack of spatial pattern and non-infectious nature of white syndrome in our study, we suggest that white syndrome may not represent a true ‘outbreak’ in the epidemiological sense of density-dependence, in that the patterns observed in the present study are not primarily due to increased ‘pathogen transmission’ in regions of higher coral cover, as previously suggested [4]. Further studies are needed to understand such local and regional ‘thresholds’ in coral cover related to white syndrome outbreaks (e.g. [23]), and to determine factors influencing recovery of communities following white syndrome outbreaks (e.g. [46], [60]). Indeed, models of tabular *Acropora* recovery following disturbance indicate rapid recovery following coral bleaching through high settlement rates and rapid initial growth of settled colonies [33]. Finally, further research is needed to identify reefs on the GBR that are potentially resilient to outbreaks of coral disease.
